# Signaling Pathways Regulating Human Cervical Ripening in Preterm and Term Delivery

**DOI:** 10.3390/cells11223690

**Published:** 2022-11-21

**Authors:** Maciej W. Socha, Wojciech Flis, Miłosz Pietrus, Mateusz Wartęga, Martyna Stankiewicz

**Affiliations:** 1 Department of Perinatology, Gynecology and Gynecologic Oncology, Faculty of Health Sciences, Collegium Medicum in Bydgoszcz, Nicolaus Copernicus University, Łukasiewicza 1, 85-821 Bydgoszcz, Poland; 2 Department of Obstetrics and Gynecology, St. Adalbert’s Hospital in Gdańsk, Copernicus Healthcare Entity, Jana Pawła II 50, 80-462 Gdańsk, Poland; 3 Department of Gynecology and Oncology, Jagiellonian University Medical College, Kopernika 23, 31-501 Kraków, Poland; 4 Department of Pathophysiology, Faculty of Pharmacy, Collegium Medicum in Bydgoszcz, Nicolaus Copernicus University, M. Curie- Skłodowskiej 9, 85-094 Bydgoszcz, Poland

**Keywords:** cell signaling, pregnancy, cervical ripening, proliferation, induction of labor, differentiation

## Abstract

At the end of gestation, the cervical tissue changes profoundly. As a result of these changes, the uterine cervix becomes soft and vulnerable to dilation. The process occurring in the cervical tissue can be described as cervical ripening. The ripening is a process derivative of enzymatic breakdown and inflammatory response. Therefore, it is apparent that cervical remodeling is a derivative of the reactions mediated by multiple factors such as hormones, prostaglandins, nitric oxide, and inflammatory cytokines. However, despite the research carried out over the years, the cellular pathways responsible for regulating this process are still poorly understood. A comprehensive understanding of the entire process of cervical ripening seems crucial in the context of labor induction. Greater knowledge could provide us with the means to help women who suffer from dysfunctional labor. The overall objective of this review is to present the current understanding of cervical ripening in terms of molecular regulation and cell signaling.

## 1. Introduction

The uterine cervix has two significant functions depending on the stage of pregnancy. First, the cervix ensures physical integrity so the fetus can develop in the uterus by the time delivery begins. Second, during the final days of pregnancy, the cervix softens and becomes more susceptible, allowing labor to occur.

The physiological parturition consists of two major phases. The first preparatory phase, called “cervical ripening”, starts during the final days of pregnancy. It consists of a series of complex biochemical pathways that lead to the rearrangement of the cervical extracellular matrix. As a result, the cervix relaxes and significantly softens. The second phase is known as “active labor”. During this stage, the cervix dilates in response to systolic uterine activity. This allows the cervix to readily pass the presenting part of the fetus. Both phases are highly conjugated, and disturbances during any of them can lead to the failure of vaginal delivery.

Over the years, strategies have been developed for labor induction. The protocol mainly involves a cervical ripening agent (for the first phase induction) followed by oxytocin/amniotomy as a next step.

The whole ripening process is a complex process derivative of enzymatic breakdown, inflammatory process, and endocrine regulation. Despite a tremendous amount of research over the years, it still seems poorly understood. The role of specific factors in cervical ripening has been described. The cervical remodeling is a derivative of the reactions mediated by those factors. However, we still do not know if one dominant factor (or pathway) is responsible for initiating the complex biochemical changes. Furthermore, if so, what are its regulations? A pivotal question is how one small piece of the uterus, the cervix, remains firm during pregnancy and opens sufficiently during labor to allow the fetus to be delivered. In summary, cervical ripening is a multifactorial process mediated by endocrine factors, inflammatory response, and other molecular changes.

Over 20% of pregnant women undergo induction of labor [1]. Almost half of them require a ripening agent because of an unfavorable cervix. Prostaglandins, progesterone antagonists, mechanical devices, and other methods have been used to ripen the cervix with varying efficacy and safety profile rates. However, according to the latest studies, no cervical ripening method seems superior, considering safety outcomes and efficiency [2].

Therefore, a precise understanding of the processes that lead to cervical ripening is crucial for developing more effective and safer methods of artificial induction of labor.

## 2. Anatomy and Physiology of Cervix

The human cervix is the inferior part of the uterus. It is located between the rectum and bladder. It develops (along with uterus and vagina) from Müllerian ducts [3]. In nulliparous women, the cervical length corresponds to the uterine corpus length. However, in multiparous women, the uterine corpus is approximately twice as long as the cervix. Moreover, the multiparous cervix is more bulbous and bigger in comparison to the nulliparous cervix [4]. The cervix can be divided into a supravaginal portion and a lower portion. The cervical canal runs between the internal and external os.

The endocervical canal is lined by a tall columnar epithelium containing a large number of glands [5]. The epithelium contains a single layer of cells that produces mucus composed of water, ions, enzymes, plasma proteins, and mucin glycoproteins [6]. In a nonpregnant state, the cervical epithelium provides a structural and immunoprotective barrier provided by the production of cytokines, chemokines, antimicrobials, and pattern-recognition receptors. The properties of cervical mucus undergo significant changes during the menstrual cycle. During the proliferative (estrogen-dependent) phase, cervical mucus becomes watery with high ionic concentration. Those changes permit sperm movement into the upper reproductive tract. During the luteal phase (corpus luteum-dependent), highly viscous mucous forms a barrier to prevent sperm invasion [6,7]. During pregnancy, the epithelium proliferates significantly. Cervical glands produce thick, highly viscous mucus, forming a plug filling the endocervical canal (Kristeller’s plug). The plug is present till term and provides a physical and immunological barrier function [7].

The cervix is formed mainly by connective tissue, smooth muscle, and ground substance and is infiltrated by lymphatic vessels and nerves [6]. The cellular element comprises mast cells, fibroblasts (which can synthesize and degrade the significant extracellular matrix parts), and wandering cells. The main component of the cervix is connective tissue with only approximately 10–15% of smooth muscle [5]. The latest studies suggest that external cervical os consist mostly of collagen fibers and approximately 10% of smooth muscle randomly scattered in the tissue. On the other hand, the internal os contains a significant amount of smooth muscle cells that are circumferentially organized in sphincter-like patterns [8]. It seems that oxytocin can stimulate internal os contraction significantly more forcefully than external os [8]. This may suggest the presence of an organized sphincter in the internal os of the cervix, which is actively involved in the functioning of the cervix during pregnancy.

The extracellular matrix (ECM) contains a large amount of collagen—mainly types I and III [5]. Additionally, there is a small amount of type IV collagen [9]. Collagen is one of the major components of the cervical ECM. Collagen fibers are linked together by covalent cross-links, making them more stable. These cross-links provide collagen stability and are responsible for maintaining resistance to degradation [9]. Because of its cross-linked, three-dimensional structure, it contributes greatly to the stiffness of the cervix. Cervical collagen fiber rearrangement is known to play a key role in the cervical ripening process [10]. Collagen fibrils bind strongly to the glycosaminoglycan (GAG) anionic groups that act as cement. A noticeable decrease in the mRNA of cervical type I collagen has been reported in the third trimester [11]. This may suggest that this significant decrease is associated with cervical ripening.

Cervical stroma (the ground substance) is made of GAGs. GAGs contain a high amount of sulfate groups, which makes them highly hydrophilic [9]. These hydrophilic molecules maintain tissue hydration and strength. They include chondroitin sulfate, dermatan sulfate, heparan sulfate, keratan sulfate, and hyaluronan. Chondroitin sulfate and dermatan sulfate are to most concentrated GAGs in the cervix [9,12]. The diversity of GAGs is regulated by the specific type of glycosaminoglycan (GAG), degree of sulfation, size of the GAG chains, and ability to bind collagen [13]. In addition, the GAG chains create covalent bonds with the protein core (except hyaluronan acid)—creating proteoglycans.

Proteoglycans have the ability to bind collagen fibers. This close association plays a pivotal role in providing cervical tissue strength. Therefore, it seems obvious that changes in proteoglycan arrangement will have profound effects on collagen binding stability. There are three main types of proteoglycans: biglycan (PG-S1), decorin (PG-S2), and chondroitin (PG-L).

Elastin fibers are also a component of the uterine cervix [14]. They consist of elastin polymer cross-linked into microfibrils. In the nonpregnant cervix, elastic fibers show long, straight integrated fibers. During pregnancy (with a peak at the end of gestation), the elastin fibrils become shorter and more dispersed [15]. Both elastin fibers and collagen fibers are influenced by steroid hormones [16]. Studies conducted on mice indicate that progesterone induces collagen and elastic fibers synthesis. Moreover, estrogen regulates genes participating in fibers synthesis, as well as elastic fiber processing and assembly [16]. We believe that progesterone and estrogen may have a strong impact on elastogenesis.

## 3. Cervical Ripening

As mentioned before, the cervix undergoes significant changes during the final days of pregnancy. These changes can be broadly defined as the rearrangement of the cervical extracellular matrix. The greatest intensity of occurring changes can be observed at the end of gestation. As a result of these changes, the cervix transforms from closed and rigid to soft and susceptible.

**Figure 1:**
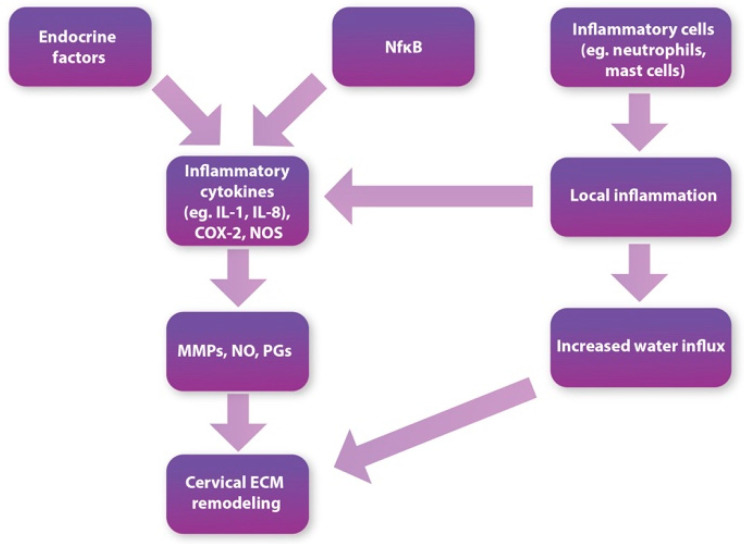
Diagram showing an outline of the changes occurring during cervical ripening.

In cervical ripening, the main histological and biochemical changes are collagen- and elastin-fiber degradation, increased fluid influx, and the development of a local inflammatory response (Figure 1). In the following section, we would like to review the histological events occurring in the cervix during its remodeling.

### 3.1. Glycosaminoglycans

Cervical remodeling is characterized by increased hyaluronan synthesis, decreased expression of collagen assembly genes, and increased distribution of inflammatory cells [17]. There is a significant increase in the cervical synthesis of glycosaminoglycan hyaluronan (HA). Cervical HA levels remain low during pregnancy and gradually increase during parturition [18]. HA is the predominant GAG in the cervical stroma at the onset of labor. It results from increased transcription of hyaluronan synthase 2 (HAS2) [19]. High HA concentration attracts water molecules and increases hydration, leading to collagen- and elastin-fiber dispersion. There is an almost 70% reduction in cervical collagen concentration at term [12]. As a result, collagen fibers become more widely spread out.

Interestingly, the total amount of proteoglycans decreases, and there is an increase in the PG-S1 and PG-L. Those proteoglycans bind less strongly to collagen fibers [20]. Moreover, there is a change in GAG composition. This strongly suggests that during the whole ripening process, there is a significant loosening of previously tightly packed collagen fibers.

### 3.2. Matrix Metalloproteinases

Matrix metalloproteinases (MMPs) secreted by stromal cells, fibroblast, inflammatory cells, and smooth muscle are believed to play a pivotal role in the cervical ripening process [21]. Macrophages and neutrophils are known sources of leukocyte collagenase (MMP-8) and collagenase-1 (MMP-1) [22]. They are the prime performers of cervical remodeling thanks to their ability to decompose collagen fibers in cervical stroma. MMPs can also digest other ECM components such as proteoglycans, laminin, and fibronectin. MMPs are synthesized as prepro-enzymes (zymogens) and secreted as inactive forms. MMPs become the active form of enzymes after proteolytic cleavage. One of the features of MMPs is that genes responsible for matrix metalloproteinase (MMP) expression can be induced by various factors such as cytokines, chemical agents, and growth factors. Moreover, MMP gene expression may be inhibited by factors such as glucocorticoids, progesterone, and retinoic acid [23]. Various MMPs are involved in the cervical ripening process: MMP-1, gelatinase A (MMP-2), MMP-8, and gelatinase B (MMP-9). MMP-1, MMP-2, and MMP-8 degrade collagen types I, II, and III, while MMP-9 cleaves collagen type IV [21,23]. The digestion process causes changes in collagen structural organization—from densely packed large fibrils with little intervening ECM material to small, more dispersed, and randomly oriented bundles of collagen. It must be mentioned that collagen is not completely disassembled during that process. The MMPs’ concentration and activity have been shown to increase in the lower uterine segment during labor [24]. Moreover, collagenolytic activity has been shown to increase in cervical stroma during cervical ripening [25]. Research has shown that nitric oxide (NO)—a potent cervical ripening agent—can increase the activity of specific MMPs [24]. The activity of MMPs can be tightly regulated by their specific inhibitors, such as β2-macroglobulin or tissue inhibitor of metalloproteinase (TIMP). The shape of TIMP looks like a wedge that binds into the active-site slot of an MMP and thus can directly affect the level of MMPs [26]. TIMPs have the ability to inhibit the proteolytic activity of MMP-2,9 [26]. The latest research indicates that TIMPs are present in a pregnant cervix [27]. The above statements indicate the presence of an activity regulation mechanism that can affect levels of MMPs. Theoretically, there is a possibility of using TIMPs as a collagenolysis inhibitor to inhibit cervical ripening in preterm delivery. However, further studies evaluating the exact role of TIMPs in cervical ripening are required.

### 3.3. Inflammatory Process and Immune Cells

Cervical ripening corresponds in many aspects to the inflammatory process. The inflammatory reaction can be triggered by infection, trauma, chemical agents, or hypersensitivity reaction. Many of the mediators and enzymes involved in regulating the acute inflammatory response are also strongly involved in regulating the cervical ripening process. Local vasodilatation associated with increased vascular permeability can be observed during the ripening process [9]. Increased vascular permeability leads to an increase in water and inflammatory cells influx. As a result, marked edema of the cervical tissue can be observed in the cervical stroma.

Many studies have reported the presence of various immune cells in the cervix (as well as in fetal membranes, uterine muscle, and decidua) during the last days of gestation [28]. There is a noticeable large influx of neutrophils, macrophages, and mast cells in cervical tissue during the last days of gestation. The spreading of granulocytes is achieved by chemokines and cellular adhesion molecules [29]. The mRNA level of C-X-C motif chemokine ligand 8 (a strong neutrophilic chemoattractant) is significantly higher in women during term labor [28]. Neutrophils are known for their pivotal role in cervical ripening and labor by releasing proinflammatory cytokines and secreting (MMPs) [30,31]. Macrophages also are among the cells that participate in the process of cervical ripening. They are significant because of the products they secrete, such as MMPs, interleukins (IL-1, IL-6), tumor necrosis factor-α (TNF-α), and NO [32].

Interestingly, mast cells (MCs) are important immunomodulating factors during late gestation and parturition [33]. They secrete a variety of mediators, such as histamine, serotonin, heparin, proteoglycans, proteases, leukotrienes, and PGs [33]. Moreover, due to mast cells degranulation, there is a significant increase in the concentration of interleukins: IL-1β, IL-3, IL-6- and TNF-α [34]. MCs are also an important source of adhesion molecules and chemokines [35].

Interestingly, histamine (secreted by MCs) has been shown to cause myometrial and cervical smooth muscle contractility [36]. In addition, histamine stimulates the phospholipase C signaling pathway in myometrial smooth muscle cells through H1 receptors [37]. In addition, allergen-stimulated mast cell degranulation induces preterm labor in guinea pigs via a type 1 hypersensitivity reaction [38]. This supports the hypothesis that there is a link between allergy and preterm birth. Furthermore, treatment of guinea pigs with histamine H1 receptor antagonist can prevent allergen-induced preterm labor [38]. The above statements suggest that histamine may play a significant role in both term and preterm labor and may be associated with type 1 hypersensitivity and labor. However, this issue requires further research to fully delineate the potential role of histamine in the cervical ripening process.

## 4. Regulation of Cervical Ripening

During cervical ripening, there is a significant accumulation of substances such as nitric oxide (NO), prostaglandins (PGs), pro-inflammatory cytokines, and metalloproteinases (MMPs). These substances (secreted by a variety of cells) are the main players responsible for maintaining the correct course of biomolecular changes occurring during the cervical ripening process. In the following sections, we would like to discuss the role of individual factors (exogenous and endogenous) that significantly regulate the course of cervical ripening. have subjectively ordered the following factors influencing cervical ripening according to their importance in the whole process.

### 4.1. Inflammatory Cytokines

The presence of inflammatory cytokines can be seen in the cervix during its remodeling. The vast majority are proinflammatory cytokines such as IL-1, IL-6, IL-8, granulocyte colony-stimulating factor (G-CSF), TNF-α, and leukemia inhibitory factor (LIF) [21,39]. As mentioned above, cervical ripening resembles an inflammatory response in many aspects. This suggests that cytokines play one of the major roles in cervical remodeling and parturition.

Cytokines are a large heterogenous group of proteins and polypeptides (usually containing 100 to 200 amino acids) responsible for regulating cell–cell interactions with various pleiotropic effects in various organs. The biological effect depends on the cytokine and the cell being involved. They can affect cell activation, division, apoptosis, or movement (chemokines). In addition, they are able to act via autocrine, paracrine, or endocrine routes [40]. The classification of cytokines includes interleukins (product of white cells), monokines (product of mononuclear cells), lymphokines (product of lymphocytes), interferons, colony-stimulating factors (CSF), and growth factors [41]. Cytokines are produced in the cervical tissue, fetal membranes, decidua, and uterine smooth muscles.

#### 4.1.1. Interleukins

There is a significant increase in the concentration of proinflammatory cytokines (IL-1, IL-6, IL-8, TNF, and granulocyte-colony stimulating factor (G-CSF)) and a decrease in the concentration of anti-inflammatory cytokines (IL-4 and IL-10) during cervical ripening [42,43].

IL-1 is an essential cervical ripening regulator. There are two major isoforms of interleukin-1: IL-1α and IL-1β. They both have a similar biological effect and are recognized by the same receptors. IL-1 can upregulate cyclooxygenase-2 (COX-2) and downregulate prostaglandin dehydrogenase (PGDH) [44]. Additionally, IL-1β stimulates the expression of prostaglandin G/H synthase (PGHS)-2 (an inducible form of the enzyme, which is a crucial modulator of the prostaglandin (PG) synthesis pathway) [45]. Thus, IL-1 acts on COX-2 to produce prostaglandins (PGs) and decreases the metabolism of PGs. Moreover, IL-1 stimulates the production of other proinflammatory cytokines, such as IL-6, IL-8, and TNF [46]. Apart from affecting cyclooxygenase and other inflammatory cytokines, IL-1 (both α and β) enhances the expression of MMP-1, MMP-3, and MMP-9 and downregulates the expression of TIMPs [47]. Finally, IL-1 stimulates fibroblasts to produce a significant amount of HA [48]. Vaginal application of suppositories with IL-1 to pregnant rabbits caused softening and dilatation of the cervix compared with the control group [49]. Considering all the above, IL-1 plays a major regulatory role in the inflammatory response during cervical ripening and may be used as a cervical ripening agent as well. However, the above statement requires further clinical studies with suitable IL-1 donors, which can be safely administered.

IL-8 is a pivotal regulator of cervical ripening. It is synthesized mainly by monocytes, fibroblasts, macrophages, neutrophils, and chorio-decidual cells [50]. Synthesis and secretion of IL-8 is significantly increased at term in cervical tissue, and the concentration of IL-8 in the cervix and lower uterine segment increases with the cervical ripening process [51]. IL-8 is a potent neutrophil chemotactic agent—it also promotes neutrophil activation [46]. Moreover, IL-8 can significantly increase vascular permeability, subsequently increasing inflammatory cell and fluid influx. IL-8 can influence cervical MMP activity by stimulating the release of MMP-8 and MMP-9 from neutrophils [52]. IL-8 not only induces infiltration of the cervical stroma by neutrophils but also increases the release of neutrophilic proteases. [53]. In addition, IL-8 can stimulate fibroblasts to produce HA [5,9]. One of the factors affecting IL-8 activity is progesterone. Research has shown that progesterone suppresses IL-8 production in endometrial cells [54]. This agrees with the statement that the functional progesterone withdrawal occurs at the end of gestation. Studies show a close relationship between IL-1 and IL-8. Vaginal administration of IL-1 led to a significant increase in IL-8 concentration in the cervix [55]. It seems that IL-1 upregulates IL-8 production in the cervical stroma. Additionally, IL-8 and prostaglandin E2 (PGE2) (which synthesis is enhanced by IL-1) have been found to act synergistically—PGE2 reducing the concentration of IL-8 required to cause neutrophil invasion [56]. This suggests that there is a strong relationship between IL-1 and IL-8 in which both of these cytokines are highly interdependent. It turns out that IL-8 can be used as a cervical ripening agent. Vaginal administration of suppositories with IL-8 to pregnant rabbits revealed a marked softening and dilatation of the cervix [57]. Taking the above into consideration, we believe that IL-8 strongly contributes to the cervical remodeling process.

#### 4.1.2. Tumor Necrosis Factor

Another factor that appears to be involved in cervical ripening is TNF-α. Tumor necrosis factor is a potent paracrine and endocrine mediator of immunological and inflammatory functions involved in various systemic processes from carcinogenesis to cervical ripening. It is also known to regulate the growth and differentiation of various cells. The main sources of TNF-α are mostly monocytes and macrophages [58]. A noteworthy concentration increase in TNF can be observed in the cervix at term. TNF-α upregulates secretion of phospholipase A2 and protein kinase C (a key enzyme involved in the synthesis of MMPs), suggesting that TNF-α stimulates PG and MMP production [46]. Additionally, TNF-α induces MMP-1, MMP-3, and MMP-9 mRNA expression [59]. Considering the above, it seems that TNF-α can alter cervical tissue composition.

### 4.2. Apoptosis

The length of the pregnancy is relatively constant. That is why the idea of genetically determined cell death triggering parturition is appealing. The remodeling of collagen fibers and other extracellular matrix components during cervical ripening seems to be quite understood. However, the mechanism initiating this whole process is still unclear—little is known about the role of cervical smooth muscles. Research shows that in rats, there is a noticeable increase in apoptosis of cervical smooth muscle cells at term [60]. A gradual increase in the number of dying smooth muscle cells can be observed as pregnancy advances, which correlates with cervical softening. It seems that the programmed apoptosis of cervical muscle cells could play a role in the cervical maturation process. Moreover, TNF-α and glucocorticoids (which are involved in the regulation of cervical softening) have the ability to regulate cell apoptosis. Another critical factor, the importance of which is constantly growing, is oxidative stress. Research revealed that exposure of cervical cells to the reactive oxygen species inducer causes cell cycle arrest and promotes apoptosis [61]. It is clear that cell apoptosis occurs in the cervix at term. However, it may respond to other events occurring during cervical ripening rather than act as an initiating factor.

### 4.3. Hyaluronic Acid

As mentioned earlier, a major increase in the concentration of HA can be observed in cervical stroma prior to parturition, and, at the onset of labor, HA is the predominant GAG in the cervix [62]. HA is a polymer made up of repeating disaccharides (D-glucuronic acid and β-1,3-N-acetylglucosamine-β-1,4). Thanks to its specific spatial structure, HA gains the ability to form solutions with high viscosity and elasticity that provide space-filling functions, serve as a substrate for assembling proteoglycans, and promote cell movement [19]. Hyaluronic acid is synthesized by three hyaluronan synthase isoenzymes—HAS1, HAS2, and HAS3. The dominant form in cervical tissue is HAS2. A balance between HA synthesis and catabolism is crucial to maintain normal cell function. Major hyaluronidase (HAase) isoenzymes that degrade HA are hyaluronidase 1 and 2 (Hyal 1 and Hyal 2). The activity of HAase greatly increases during labor. It is suggested that PGs, steroids, and peptide hormones could control HA synthesis. HA concentration significantly increases in the cervix primed with PGE2, relaxin, and antiprogesterone [63,64,65]. HAS2 transcription is upregulated at the end of pregnancy, and its mRNA expression is greater than other HAS expression [62]. It seems that HAS2 could be a key modulator of HA concentration at term.

HA has a significant impact on the cervix. First of all, HA has a high affinity to water molecules and therefore causes the influx of water to the cervical stroma at term, leading to collagen fiber dispersion. Second, HA stimulates the fibroblast-dependent secretion of collagenolytic enzymes and upregulates IL-8 production [18]. Another great feature of HA is that the chemotactic response of neutrophils markedly increases in the presence of HA [66]. However, HA functions seem dependent on their size and structure. It has been reported that an increase in concentration of HA is followed by its degradation (mediated by hyaluronidase) into low-molecular-weight hyaluronic acid at the onset of labor. A significant correlation between HA and HAase activity can be observed. This low-molecular-weight HA induces cytokine production, neovascularization, and MMP production and upregulates iNOS, which are strong cervical ripening mediators [17,18,67,68]. Furthermore, it has been reported that low-molecular-weight HA induced cervical ripening in rabbits [18]. Interestingly, low-molecular-weight HA production can be stimulated by IL-1 [69]. We suggest that HA causes water influx and mediates the neutrophil chemotactic response. Then, hyaluronidase and reactive oxygen species decompose HA into low-molecular-weight HA, significantly enhancing cervical ripening. These reports suggest that low-molecular-weight HA plays a pivotal role in the cervical remodeling process. It is suggested that fibroblasts and macrophages are the main sources of hyaluronidase during cervical ripening. However, this phenomenon requires further research.

Apart from HA, it seems that HAase plays an even greater role in the cervical ripening process. Research revealed that hyaluronidase could be used as an effective cervical ripening agent. It turns out that intracervical application of hyaluronidase causes cervical ripening. Moreover, a significantly shorter duration of labor and a larger chance of vaginal delivery was observed [70,71,72]. Interestingly, the use of HAase for cervical ripening is not associated with uterine contractions and uterine myometrial hyperstimulation [73]. It seems that using hyaluronidase is a safe, highly effective, low-cost method for cervical ripening.

Considering the above, HAase could be a potentially superior agent for the cervical remodeling process compared to the currently available methods. Therefore, we suggest that HAase and low-molecular-weight HA play a great role in cervical tissue remodeling.

### 4.4. Erythropoietin

Interestingly it is postulated that erythropoietin (EPO) may play a role in cervical ripening and preterm labor. EPO is known for its participation in hypoxia-induced erythropoiesis. It binds to the EPO receptors (EPORs), which form disulfide-linked homodimers, and selectively activates JAK2 kinase, phosphorylating the Jak2 protein. This pathway activates various signaling cascades, such as STAT3 activation and AKT or ERK1/2 pathways [74]. Apart from in the kidney, EPO is expressed in the placenta and myometrium. Preterm labor is often associated with intrauterine infection. LPS-induced preterm parturition leads to the increased expression of proinflammatory cytokines, nuclear factor kappa-light-chain-enhancer of activated B cells (NF-κB), PGs, and NO, which are also involved in the cervical ripening process [75]. The latest research shows that the application of EPO to mice with inflammatory-associated preterm delivery has promising effects. It was shown that EPO may prevent preterm birth by inhibiting the expression of proinflammatory cytokines such as: IL-1, IL-6, and TNF-α. Additionally, EPO decreased PG concentration, inducible nitric oxide synthase (iNOS) expression, and NF-κB activity [76]. Clearly, EPO has a noticeable effect on parturition and cervical ripening.

Taking all into consideration, it seems that EPO could be used as a tocolytic agent and inhibitor of premature cervical ripening in preterm birth. However, the side effects that may be associated with EPO should be considered. Due to its ability to stimulate erythropoiesis, EPO significantly increases the risk of thromboembolic complications. Having said that, further studies evaluating the role of EPO in cervical ripening are required.

### 4.5. Mechanical Factors

Cervical ripening is a process that consists not only of biochemical and molecular pathways but also of the mechanical factors involved. Presenting part of the fetus exerts passively on the cervix by pressure effects. It leads to connective tissue changes during ripening. Apart from the mechanical distension of the cervix, the fetus, during its descent, causes stretching of fetal membranes and myometrial cells, leading to increased secretion of IL-8, PGE2, and collagenase activity. Those factors have a significant effect on cervical maturation [77,78]. Overstretching of the uterus and fetal membranes in the case of polyhydramnios and multiple pregnancies can be accompanied by premature cervical ripening and preterm delivery.

Apart from the soluble signaling molecules that are crucial for normal cell function, mechanical signaling has been shown to play an important role in cell homeostasis. ECM, which is a dynamic structure of enzymes, proteins, cytokines, etc., may transduce information to cells via stress on cellular membranes affecting stretch-sensitive ion channels and changes in biopolymers. Research shown that cyclic mechanical stretch can increase HA expression in fibroblasts which enhances the influx of water to the cervical stroma leading to collagen fibers dispersion [79].

Therefore, it appears logical to suggest that passive mechanical stretching by presenting part of the fetus impacts cervical ripening. However, this issue requires further research.

### 4.6. Endocrine Regulation

As mentioned earlier, during cervical ripening, the extracellular matrix of the cervix is rearranged. The entire process is the result of many interwoven biochemical pathways that depend on hormonal regulation. In the following sections, we would like to discuss the endocrine regulation of the cervical ripening process.

#### 4.6.1. Relaxin

Relaxin (RLX), among other significant effects it has on the entire organism during pregnancy and parturition, has been shown to assist in reorganizing cervical connective tissue during the maturation process. RLX is a member of the insulin family hormones. It consists of two peptide chains (A and B) linked together by two disulfide bonds. The main source of relaxin during pregnancy are decidual cells. RLX serum concentration peaks in the first trimester and remains constant throughout pregnancy, but at the end of pregnancy concentration of RLX increases in cervical tissue [80]. The impact of RLX on the cervix is mainly manifested by its influence on collagen, GAGs, and MMPs. The action of relaxin causes a decrease in cervical collagen concentration and an increase in HA concentration. Additionally, RLX up-regulates MMPs activity which subsequently can cleave collagen cross-links [81,82,83].

Research revealed that vaginal administration to pregnant women of recombinant human RLX caused no effect as a cervical ripening agent [84,85]. However, the administration of relaxin with estrogen to pregnant rats at term resulted in changes similar to those for cervical ripening [86,87]. We suggest that estrogen and relaxin appear to have a synergistic effect on cervical tissue remodeling. Relaxin seems to maintain the cervical ripening process in the presence of estrogen.

The impact of relaxin on myometrial cells is still debatable. Relaxin has been shown in rats and pigs to induce myometrial quiescence inhibiting spontaneous and oxytocin-induced contractions [82,88]. However, the same effect could not be demonstrated in pregnant human myometrial cells [89]. RLX acts on myometrial cells via its specific plasma membrane receptor (RFXP1). There are several post-receptor signal transducing mechanisms. First, RLX causes the opening of the plasma membrane K^+^ channels, leading to cell hyperpolarization. Second, stimulation of the RFXP1 receptor leads to the generation of cyclic adenosine 3′5′ monophosphate (cAMP). cAMP catalyzes the phosphorylation of the catalytic subunit of the myosin light chain kinase (MLCK). Phosphorylation of MLCK inhibits its capacity to form the active MLCK—Ca^2+^- calmodulin complex required for uterine smooth muscle contraction [90]. In conclusion, RLX acts via the RFXP1 receptor and causes the relaxation of uterine smooth muscle. Recent research has shown that human myometrial cells respond to relaxin by increasing cAMP concentration but with minor effects [89].

We suggest that differences in response to relaxin among different species may be due to the different values of RLX concentration at the end of pregnancy or some additional regulatory factor that remains unknown. Considering all the above, relaxin plays a role in pregnancy and parturition. However, a detailed understanding of its function requires further research.

#### 4.6.2. Corticotropin-Releasing Protein Hormone

Corticotropin-releasing protein hormone (CRH) is a main regulatory factor of the hypothalamic–pituitary–adrenal axis (HPA). CRH stimulates adrenocorticotrophin (ACTH) release from the pituitary gland. ACTH stimulates the excretion of glucocorticoids from the adrenal glands. A negative feedback loop exists—glucocorticoids can inhibit CRH and ACTH release from glands. CRH is produced by the fetal hypothalamus, maternal hypothalamus, and syncytiotrophoblast cells. Placental CRH and hypothalamic CRH have identical sizes and biological activity. CRH levels rise gradually during pregnancy until the final weeks of gestation, when a dramatic sharp rise occurs [91]. CRH activity is regulated by corticotropin-releasing hormone-binding protein (CRHBP). During the final days of gestation, CRHBP level decreases, CRH levels increases, and CRH activity enhances [92]. In addition, CRH can stimulate myometrial activity. A variety of factors are shown to regulate CRH secretion and activity. TNFα, IL-1, and PGs can stimulate CRH release, whereas progesterone may inhibit CRH synthesis [93,94]. Quite apart from the CRH endocrine functions and its influence on uterine activation on the onset of labor, it is also believed that the cervix may be a target for CRH action. CRH protein and its receptors (CRH-R1 and CRH R-2) were detected in the cervical tissue with the highest levels at term [95]. There are several mechanisms by which CRH may be involved in the cervical maturation process: by enhancing the PGs production, upregulating expression of iNOS, and stimulations of MMP-9 secretion [96,97]. Additionally, it seems that CRH plays a role in inflammatory events. CRH has been implicated in inducing the production of chemokines and cytokines, which causes chemotaxis of leukocytes and subsequent inflammation. CRH promotes inflammation mainly via activation of NF-κB, which seems to be a key mediator of the inflammatory response [98]. CRH binds to its receptor (CRH-R1), coupled with Gα protein. Activation of CRH-R1 subsequently enhances the activation of adenyl cyclase, the enzyme that catalyzes the conversion of ATP to cAMP. cAMP activates cAMP-dependent protein kinase A (PKA), which activates the NF-κB complex and promotes its translocation to the nucleus to stimulate inflammatory cytokines gene transcription [99].

#### 4.6.3. Glucocorticoids and NF-κB

Placental CRH stimulates fetal ACTH secretion, which subsequently stimulates cortisol production. Glucocorticoids can promote placental CRH gene expression and thus upregulate the production of CRH [100]. As a result, there is a rise in fetal cortisol production at term. The glucocorticoid receptor (GR) can be found not only in the placenta and membranes but also in the cervical stroma. GR and the progesterone receptor share structural similarities and interact with the same hormone-responsive elements [101]. GR has two isoforms—GRα and GRβ, with the dominance of GRα. Glucocorticoids exert their anti-inflammatory functions by inhibiting the expression of cytokines, iNOS, MMPs, and COX-2. Interestingly, NF-κB has been shown to have diametrically opposed functions [102]. NF-κB (nuclear factor-kappaB) is a family of transcription factors such as cRel, p50, p52 and RelA/p65, RelB. Activated NF-κB gains the ability to translocate to the nucleus and subsequently activate gene transcription, thus enhancing the expression of inflammatory mediators such as IL-1, IL-8, IL-6, iNOS, and COX-2, which are crucial for cervical ripening. A significant decrease in GR levels in the cervical stroma at term with an increase in NF-κB can be observed [103]. We suggest that changes in GR levels and an increase in NF-κB activity in the cervical stroma may be closely related. It is very possible that the reduction in glucocorticoid activity contributes to the activation of NF-κB, which then gains the ability to increase the transcription of genes coding for factors regulating the cervical remodeling process.

Interestingly, anti-inflammatory prostaglandin 15-deoxy^Δ^12,14-prostaglandin J2 (15dPGJ2) has an ability to inhibit NF-κB action. It is obvious that inflammatory response plays a key role in labor (both term and preterm). Theoretically, targeting key inflammatory pathways may be beneficial in reducing the risk of spontaneous preterm birth. Especially, targeting NF-κB activity could result in inhibition of COX-2 activity and reduce synthesis of pro-inflammatory cytokines, which are strongly involved in cervical ripening. However, further studies are required for developing novel targets and indications for anti-inflammatory drugs usage in the field of preterm labor prevention [104].

#### 4.6.4. Dehydroepiandrosterone 3-Sulfate and Interleukin-8

Besides stimulating cortisol excretion, ACTH is also known to stimulate dehydroepiandrosterone 3-sulfate (DHEA-S) production, which seems to be associated with cervical ripening. It is mainly produced in adrenal glands; then it is converted in the fetal liver to 16-hydroxy-DHEA-S and travels to the placenta, where it is converted into estrogen. There is a significant increase in DHEA-S concentration in the cervical stroma at term [105]. It seems that there may be a strong relationship between DHEA-S and IL-8. Vaginal application of DHEA-S and IL-8 have led to a greater increase in MMPs activity, an increase in neutrophil influx, and a decrease in total collagen concentration in cervical tissue compared to the use of IL-8 alone [106]. This shows that DHEA-S might have a synergistic effect on IL-8 action. Additionally, it was shown that DHEA-S can stimulate the expression of IL-8 and IL-8 receptors and enhance HA production in the cervical stroma [107,108]. As mentioned before, HA is a strong IL-8 synthesis stimulator. Moreover, HA-induced IL-8 synthesis and the IL-8 receptor expression are stronger when DHEA-S combines with HA [109]. Taking all the above into consideration, we suggest that DHEA-S modulates the autocrine system of IL-8 not only by stimulating the expression of IL-8, IL-8 receptor, and HA but also by potentiating the effects of all of the mentioned factors.

#### 4.6.5. Estrogen and Insulin Growth Factor-1

Estrogen is the major circulating hormone of human pregnancy. The placenta is the main source of estrogen, and its concentration increases along with gestational age. It is produced via the aromatization of maternal C19 androgens such as DHEA-S [110]. CRH significantly increases estrogen production and secretion. Estrogen greatly influences myometrial changes during the final days of pregnancy, allowing coordinated uterine contractions. These changes include upregulation of PG production, increased oxytocin receptors expression, increased gap junction formation in the myometrium, and upregulation of calmodulin and myosin light chain kinase. Besides the influence on myometrium, estrogen might also impact the cervical ripening process. The presence of estrogen receptors in cervical stroma has been proved. Activation of a ligand-receptor complex leads subsequently to the interaction with specific DNA sequences that induce the transcription of particular genes [111]. Therefore, estrogen may impact cervical tissue via stimulating MMPs activity and increasing neutrophilic influx (via stimulating the expression of endothelial leukocyte adhesion molecule-1). Moreover, estrogen can induce apoptosis and promote the binding of relaxin [111,112,113,114]. Therefore, estrogens may play a regulatory role in cervical ripening. However, research has shown a significant downregulation of estrogen receptors in cervical tissue at term with an almost fourfold increase in insulin growth factor 1 (IGF-1) mRNA. These changes coincided with a decrease in collagen concentration [111]. Therefore, an increase in IGF-1 mRNA could be due to high serum estrogen concentration.

We believe that IGF-1 could be a pathway for estrogen action, and estrogens likely influence cervical ripening through processes other than the direct impact on cervical tissue. In support of this theory, IGF-1 can increase estrogen receptor activity on the transcriptional level [115]. Therefore, the increased IGF-1 levels can enhance estrogen receptor activity. Therefore, it is possible that estrogen may impact cervical tissue by acting via IGF-1. However, a clear understanding of the phenomenon requires further research.

#### 4.6.6. Progesterone

It has long been known that progesterone is one of the key modulators of cervical maturation. Its effect on the cervix is manifested by inhibiting cervical ripening and maintaining pregnancy [9]. It is also known that the application of progesterone antagonists (mifepristone) induces cervical ripening [116,117]. According to the research, mifepristone can reduce the likelihood of cesarean sections being performed for failed induction of labor [118]. In mid-pregnancy, progesterone is dominant over estrogen and inhibits the production of various inflammatory mediators, thus keeping the cervix closed and rigid. Progesterone can influence the cervix through a variety of pathways. It appears that progesterone inhibits cervical collagen decomposition. It inhibits the production of MMP-1, MMP-3, and MMP-9 (which are mediated by IL-1) and upregulates the production of TIMPs [119]. Progesterone also inhibits the production of IL-8, which is a strong chemotactic activator [120]. Finally, progesterone downregulates COX-2 activity and maintains PGDH activation at a high level [121]. Apart from the strict effect on the cervix, progesterone seems to be closely related to other hormones involved in the process of cervical ripening. Recently, it has been shown that progesterone may inhibit placental CRH synthesis and thus influence cervical ripening [93]. In animals, there is a significant shift from progesterone to estrogen dominance at term. It is achieved by 17-α-hydroxyprogesterone lyase (the production of which is stimulated by elevated levels of fetal cortisol), which is responsible for progesterone breakdown. However, the human placenta does not contain this enzyme, and the above process cannot occur during parturition [122]. In humans, a decrease in progesterone level is not observed during late pregnancy. The progesterone level is relatively high during pregnancy and parturition, decreasing only after delivery [123,124]. It is, therefore, necessary to ask how cervical ripening occurs despite high progesterone concentration levels. It is postulated that there is a functional progesterone withdrawal at term. We believe that changes in progesterone receptors isoforms expression are responsible for this withdrawal. Progesterone receptors (PRs) have been identified in the human cervix during pregnancy. PRs can be defined as cellular proteins that affect cellular function via genomic pathways (nuclear progesterone receptors (nPRs)) and by modulating intracellular signaling pathways (membrane receptors (mPRs)). There are two isoforms of this receptor: PR-A and PR-B [125]. PR-B has a strong transcriptional activity, whereas PR-A has minimal transcriptional activity and can repress the transcriptional activity of PR-B [126,127]. This suggests that PR-A is an endogenous repressor of PR-B-mediated transcriptional activity. PR-B contains an additional 164 amino acid sequences at the N-terminal, which each required for transcriptional activation of progesterone [128]. Research showed a significant downregulation in PR activity in cervical tissue at term with an increase in the PR-A:PR-B ratio [111,129,130]. It seems that genomic progesterone responsiveness is related to the PR-A:PR-B ratio. We suggest that when the PR-A:PR-B ratio increases, the local activity of progesterone declines. Subsequently, the inhibitory effect of progesterone is diminished, and there is an upregulation of a range of “pro-labor” genes (such as inflammation-related factors) normally repressed by progesterone. Interestingly, NF-κB may be involved in that process. Research shows that NF-κB represses PR-dependent transcription, and PRs can repress NF-κB-dependent transcription as well [131,132]. Activation of NF-κB leads to its translocation to the nucleus, where it can bind specific target genes and increase the expression of COX-2, IL-8, IL-6, IL-1, and NOS [131]. It seems that NF-κB may contribute to progesterone withdrawal by repressing PRs. Additionally, NF-κB can upregulate the expression of specific cervical ripening mediators. Another interesting issue explaining the mechanisms underlying progesterone withdrawal in cervical tissue is the increase in local progesterone metabolism during cervical remodeling. Human cervical tissue contains 20 α-hydroxysteroid dehydrogenase (HSD) and 5 α-reductase. These enzymes maintain the conversion of progesterone to its inactive form. The activity of HSD is upregulated by aldo–keto reductases family (AKR). Interestingly, IL-1 has an ability to increase the expression of AKR enzymes and thus to promote local progesterone metabolism in human cervical tissue. We believe that when the level of IL-1 increases during local inflammatory response, the local progesterone metabolism is gaining momentum, allowing cervical ripening to occur [133,134].

Taking all of the above into consideration, progesterone appears to be closely related to other factors that regulate cervical remodeling. However, further research is needed to fully understand the metabolic pathways influencing the role of progesterone in the cervical ripening process.

#### 4.6.7. Oxytocin

Oxytocin (OXT) is widely known to be a key player in parturition and is crucial for initiating and maintaining proper parturition. OXT precursor is synthesized in the supraoptic and paraventricular nucleus. Subsequently, after enzymatic cleavage, OXT is stored and secreted (along with vasopressin) in the posterior lobe of the pituitary gland (neurohypophysis) [135]. Certain stimuli can increase the excitability of neurons in the paraventricular and supraoptic nucleus leading to the pulsatile release of OXT from neurohypophysis [9]. Those stimuli include breast stimulation and vaginal and cervical stretching. Stretch or dilatation of the cervix is a potent inducer of OXT secretion, mediated by neural pathways called the Ferguson’s reflex. The signal is relayed by somatic spinal afferents to A2 noradrenergic neurons in the nucleus tractus solitarii (NTS) that projects to the neurons in supraoptic and paraventricular nuclei, which enhance the OXT gene transcription [136]. Oxytocin function is strongly regulated by estrogen and progesterone. Besides affecting its receptors, OXT also stimulates the production and release of arachidonic acid from decidual cells, potentiating its effects on myometrium [137]. OXT receptors are present both in the myometrium and cervix, but their concentration in cervical tissue is very low compared to other compartments, such as the uterine corpus or decidua [138]. It is clear that OXT has a direct effect on the myometrium. Considering its mechanism of action, it seems that OXT has no clear effect on cervical composition during term pregnancy.

Finally, we would like to discuss the role of the two most important factors facilitating the proper course of cervical remodeling. Nitric oxide and prostaglandins appear to be key factors as they can potentiate and coalesce the effects of other cervical ripening mediators. Additionally, both of these substances are potent cervical ripening factors.

### 4.7. Nitric Oxide

Nitric oxide is a multifunctional particle that mediates a number of diverse physiological processes. In the human female reproductive tract, NO plays an important role in a variety of pathways, including cervical ripening. NO is a highly reactive gas molecule with a half-life of approximately 4 s. It is synthesized from L-arginine. The overall reaction consists of a two-step conversion of L-arginine to NO and L-citrulline via hydroxy-L-arginine as an intermediate [9]. This reaction is mediated by nitric oxide synthases (NOS). Three isoforms of this enzyme have been isolated. These include endothelial NOS (eNOS), neuronal NOS (nNOS), and inducible NOS (iNOS). Both nNOS and eNOS are expressed constitutively, and their activity is calcium dependent, whereas the expression of iNOS is induced by cytokines and bacterial lipopolysaccharides, independently of calcium, producing large quantities of NO for hours [139]. Nitric oxide can function as an intracellular messenger, a paracrine mediator, and a neurotransmitter. It seems that NO can affect target tissues directly and indirectly. The direct effect is manifested by activating guanylate cyclase, which stimulates cGMP production from GTP. At the same time, indirect effects include oxidation and nitration, which can lead to altered protein structure or function [140]. Nitric oxide is one of the key players in the cervical ripening process. All three NOS isoforms can be found in human cervical tissue, with the dominance of iNOS [141]. nNOS is localized in epithelial cells, eNOS in vascular endothelium, and iNOS in stromal cells. Neutrophils and macrophages are likely to be a source of NO during cervical ripening as they contain iNOS [142]. An increased concentration of NOSs with a subsequent increase in NO production during labor can be observed in the cervical tissue [143]. NO can influence cervical tissue by stimulating MMP-2 and MMP-9 release [144,145]. Interestingly, NO and PGs seem to be strongly related. Nitric oxide is known to be a powerful inducer of COX-2 [146,147]. Application of a NO donor to the human cervix induced PGs synthesis [148]. It seems that by direct stimulation of COX, nitric oxide increases PGs production during inflammation. Moreover, misoprostol (a prostaglandin E1 analog) can induce NO release (by increasing the concentration of NOS) in the uterine cervix [149]. NO and PGs can induce local vasodilatation and increase leukocyte influx to the cervical stroma [147]. It turns out that nitric oxide may also affect neutrophil chemotaxis. Research showed that NO can indirectly increase neutrophilic chemotactic response by stimulating IL-8 production [150]. As mentioned before, PGs have been found to reduce the concentration of IL-8 required for effective neutrophil invasion. The above data clearly indicates a strong relationship between NO and PGs and they can operate jointly in cervical remodeling.

We believe that COX regulation by NO shows a new strong mechanism that amplifies the course of the inflammatory response mediated by PGs. Interestingly, it seems that nitric oxide can also be involved in apoptosis. Research showed that NO (being a reactive form of nitrogen) is an apoptotic and antiapoptotic substance depending on the target cell type and NO concentration [151]. However, the exact regulatory mechanisms of apoptotic and antiapoptotic activity of NO need to be elucidated. In addition, nitric oxide can significantly inhibit cervical smooth muscle contractility (by increasing the synthesis of cGMP) [152]. Nitric oxide activity can be regulated by various factors involved in the cervical ripening process. Production of NO is enhanced by proinflammatory cytokines such as IL-1, IL-6, IL-8, and TNF [153,154]. IL-1 seems to be the strongest stimulator of NO production, among other cytokines. Nitric oxide seems to be under the influence of progesterone. Due to progesterone action, uterine NO production is increased during pregnancy, but cervical NO production is suppressed [145,155,156,157,158]. After functional progesterone withdrawal occurs, cervical NO production increases, and uterine NO production decreases.

We believe that during the pregnancy, the NO system may be partially responsible for maintaining the uterine quiescence and may be involved in the initiation of parturition subsequent to a decrease in NO production in the myometrium. Apart from its effects on the cervix and myometrium, NO significantly affects uteroplacental circulation. In human uteroplacental circulation, nitric oxide can be produced constitutively (by eNOS) and in response to shear stress. NO relaxes both placental and umbilical vessels to reserve low resistance to flow [159,160]. There is ample evidence that NO causes cervical ripening. A local application of NO donor (sodium nitroprusside) can effectively induce cervical ripening while not inducing labor in pregnant guinea pigs [157]. Moreover, the application of NO donors is associated with a significant reduction in the rate of uterine hyperstimulation compared to the use of prostaglandin analogs [161]. Finally, treatment of guinea pigs with NOS inhibitor-induced parturition significantly delayed cervical ripening, resulting in prolonged deliveries [162]. NO donors seem safe and have no major side effects on the fetus or mother [163,164]. However, the above statements require further clinical studies with a suitable NO donor, which can be safely administered.

**Figure 2:**
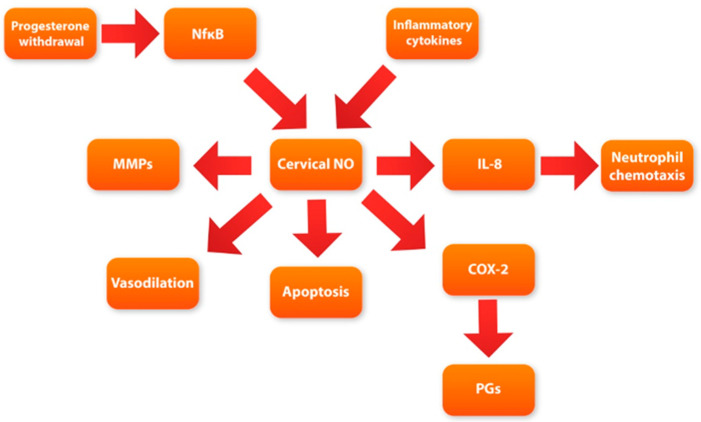
Diagram showing potential role of nitric oxide (NO) in cervical ripening process.

We believe that using ripening agents that do not stimulate uterine contractions may be much safer than other known agents. The relaxation effects of NO on the myometrium and uteroplacental vessels might be an additional benefit. Taking all the above into consideration, we suggest that nitric oxide is a crucial regulatory factor not only in remaining uterine quiescence but also in cervical ripening. It seems that NO can be a final mediator connecting other metabolic pathways (Figure 2) of cervical ripening.

### 4.8. Prostaglandins

Prostaglandins are known to have a tremendous effect on the female reproductive tract. They affect not only the cervix during its remodeling but also strongly contribute to uterine systolic activity. Prostaglandin analogs (e.g., misoprostol and dinoprostone) have been long used by clinicians for labor pre-induction [165,166,167,168]. They have proven effective and relatively safe to use with only a few adverse effects. Despite the widespread use of PGs in labor induction, their exact molecular role in cervical ripening has not been fully elucidated. PGs consist of a pentane ring with two fatty acyl chains attached to adjacent carbons. They belong to the group of eicosanoids and are derived from arachidonic acid (AA)—a 20-carbon unsaturated fatty acid that can be found in membrane phospholipids of the cell [169]. AA is liberated from membrane phospholipids via phospholipase A2 (PLA2). Arachidonic acid is converted to prostaglandin H2 (PGH2). PGH2 is subsequently converted into bioactive prostaglandins, including prostaglandin PGE2, PGF2α, PGD2, PGI2, and TXA2 [170]. This reaction is mediated by cyclooxygenase (COX). The cyclooxygenase enzyme exists as two major isoforms—COX-1 and COX-2. Despite the fact that they share the same enzymatic properties, those two isoforms are differentially regulated. COX-1 is constitutively active in various tissues, while growth factors, cytokines, and LPS induce the expression and activity of COX-2. COX-1 is mainly responsible for physiological homeostasis, while COX-2 is involved in the inflammatory response [171,172]. The activity of PGs is regulated by the local balance between the COX-2 and 15-hydroxyprostaglandin dehydrogenase (15-PGDH), which mediates the degradation of prostaglandins. PGs act via the membrane-attached prostaglandin EP receptor family, which consists of four isoforms: EP1, EP2, EP3, and EP4 [173]. They are G protein-coupled receptors that have been shown to exert various effects (including smooth muscle contractility and relaxation). EP1 and EP3 receptors facilitate smooth muscle contractility, while EP2 and EP4 promote smooth muscle relaxation. EP1 receptor is coupled with the Gq protein, and stimulation leads to smooth muscle contraction (via Ca^2+^ influx). EP2 and EP4 receptors stimulate cAMP production via the Gs protein, leading to smooth muscle relaxation. In contrast, the EP3 receptor (coupled with the Gi protein) inhibits cAMP production and promotes smooth muscle contraction [174]. The EP2 receptor is a predominant relaxatory EP subtype. All four subtypes of receptors are presented in the decidua, fetal membranes, myometrium, cervical smooth muscle, and cervical epithelium [133]. However, their expression changes during pregnancy and depends on localization and the duration of pregnancy. EP1 receptor concentration in the upper myometrium segment and overall expression of the EP2 receptor in the lower myometrium segment increases significantly at term pregnancy compared with early pregnancy. Upregulation of the EP2 receptor in the lower segment suggests that it may play a role in the relaxation of the lower segment of the uterus, allowing the fetus to deliver. EP3 receptor concentration increases in the upper myometrium segment during pregnancy [175]. The above data suggest that EP receptor expression in myometrium may play a role in the maintenance of human labor. Apart from their influence on the initiation of labor and their strong effect on uterine systoli activity, PGs seem to be important mediators of cervical ripening. The PG-induced cervical tissue remodeling appears to be mediated through the EP4 receptor, which shows maximal expression in the cervical tissue at term. Evidence suggests that EP4 activation is most likely responsible for regulating cervical tissue remodeling [176]. PGE2 appears to be the most important mediator of cervical ripening among other PGs [9]. Prostaglandins seem to promote cervical ECM changes by increasing water content and decreasing total collagen concentration [177]. Moreover, PGs stimulate GAG synthesis in cervical fibroblasts [178]. PGs strongly stimulate MMP activity, which makes it possible to cleave cross-links between cervical collagen fibrils [179]. Additionally, PGE2 can inhibit the release of secretory leukocyte protease inhibitor (SLPI), a strong inhibitor of neutrophilic function [180]. Interestingly, PGs seem to be highly involved in the inflammatory response during cervical remodeling. PGE2 stimulates the expression of endothelial adhesion molecule ICAM-1 in endothelial cells, which promotes the adhesion of leukocytes to the vascular endothelium and thus facilitates inflammatory cell infiltration [181]. Prostaglandins can upregulate IL-8 release, subsequently stimulating vasodilation and vascular permeability. It seems that PGs may facilitate neutrophil influx [180]. Cytokines such as IL-1, IL-6, IL-8, and TNF can increase the production of PGs and upregulate EP4 receptor expression [41,182].

Considering the above, we believe there is a strong relationship between prostaglandins and inflammatory cytokines, where these molecules drive each other and participate in an inflammatory response leading to cervical remodeling. Interestingly, it appears that PGs may participate in functional progesterone withdrawal. Activation of protein kinase C (PKC) by PGE2 increased expression of the PR-A isoform without affecting PR-B isoform concentration. Therefore, a significant increase in the PR-A:PR-B ratio can be observed [183]. As mentioned before, PGs act on cervical tissue via the EP4 receptor. Recent research has shown that cAMP might be a selective modulator of NF-κB action [99]. Increased cAMP concentration (because of PG action) stimulates PKA, which promotes NF-κB activity. Activation of NF-κB leads to its translocation to the nucleus, where it can bind specific target genes and increase the expression of proinflammatory cytokines and NO. Moreover, it seems that IL-1 also can be involved in progesterone withdrawal. As mentioned above, IL-1 may facilitate progesterone metabolism by upregulating the expression of the aldo–keto reductase enzyme family. Additionally, IL-1 is known to be a powerful PG synthesis inducer. We believe that when local inflammation occurs, IL-1 influences the progesterone withdrawal both by stimulating its metabolism and by increasing the synthesis of prostaglandins, which may affect the progesterone receptor ratio and thus influence the cervical ripening process. However, this phenomenon requires further studies to evaluate the exact mechanism of progesterone withdrawal.

The above finding is in keeping with the fact that PGs are known to have effects leading to cervical ripening.

Prostaglandins are widely and successfully used as a pharmacological method of labor pre-induction. The available agents are misoprostol and dinoprostone. Both substances are effective but differ in pharmadynamics. Dinoprostone, which is chemically identical to endogenous prostaglandin E2 (PGE2), is available as a cervical gel or a vaginal insert, whereas misoprostol (available as a vaginal insert) is a synthetic analog of PGE1, which has an affinity for the EP2 and EP3 receptors. Therefore, the use of misoprostol as a cervical ripening agent is associated with a much higher rate of uterine hyperstimulation than the use of dinoprostone [184].

It seems that prostaglandins are involved in cervical tissue remodeling and appear to be a link between endocrine changes in the later stages of pregnancy and inflammatory changes that occur in the cervix during its remodeling. We suggest that prostaglandins are a key player in cervical ripening. It seems that PGs play a pivotal role in the remodeling of the cervix not only by directly affecting the cervical tissue composition but also by mediating and combining various factors holistically involved in the parturition process.

## 5. Discussion

The overall objective of this review is to present the current understanding of cervical ripening in terms of molecular regulation. Here we would like to summarize the sequence of events in the female cervix during its remodeling. Cervical remodeling is very similar to the inflammatory reaction. Vasodilatation with a subsequent increase in the influx of inflammatory cells can be observed. Additionally, apoptosis of cervical smooth muscle might occur. However, its significance is still uncertain. Inflammatory cells such as neutrophils, mast cells, and macrophages are a source of MMPs, which are essential for cervical collagen fiber degradation. Moreover, inflammatory cells secrete a variety of proinflammatory cytokines, which are crucial because of their regulatory role. Regulation of the entire process by cytokines occurs at almost every stage of cervical maturation. Cytokines such as IL-1, IL-8, IL-6, and TNF-α play a pivotal role in cervical ripening by stimulating MMP secretion, reducing TIMP activity, upregulating COX-2, downregulating PGDH, and increasing NO production. Additionally, cytokines are strong chemotactic agents that subsequently enhance inflammatory response in cervical tissue. Nitric oxide is another necessary factor involved in the cervical ripening process. We believe that NO may play a key regulatory role in human parturition. It is responsible for providing uterine quiescence during pregnancy and preserving low vascular resistance in the umbilical arteries. Apart from affecting fetal hemodynamics and uterine muscle, nitric oxide is responsible for a variety of actions in cervical tissue. NO not only directly stimulates MMP action and PG production but also indirectly enhances the inflammatory response via chemotaxis and local vasodilatation. It has been shown that local application of NO donors causes cervical ripening. NO actions can be stimulated by a variety of factors, such as PGs and cytokines. However, it seems that, among other factors, NO plays a pivotal role in cervical tissue because of its multi-level action. We believe that nitric oxide is not only a powerful ripening mediator, but also a substance that enhances and amplifies the effect of other factors involved in the cervical remodeling process. Prostaglandins (mainly PGE2) may also interact in the inflammatory reaction that occurs in cervical tissue by promoting vasodilatation, increasing the expression of adhesion molecules, and upregulating IL-8 secretion. Additionally, PGs are strong NO inducers. PGE2 also contributes to collagen decomposition by stimulating MMPs production and inhibiting SLPI. We conclude that both PGs and NO bond and strengthen the inflammatory response that occurs in the cervix during the ripening process. As mentioned above, human parturition is regulated by endocrine factors, which appear to be a trigger for successful parturition. Activation of the fetal hypothalamic–pituitary–adrenal–placental axis appears to be an important factor in human parturition. Activation of CRH leads to myometrial activation, as well as to enhancing an inflammatory response via NF-κB upregulation. Additionally, CRH directly stimulates COX-2 and iNOS, which contribute to cervical tissue rearrangement. DHEA-S contributes to the inflammatory response by enhancing IL-8 action and increasing HA production. Estrogen influences cervical tissue and myometrium. Estrogen apparently prepares the myometrium for contraction by increasing oxytocin receptor expression, increasing gap junction formation, and upregulating calmodulin and myosin light chain kinase. In cervical tissue, estrogen stimulates MMP secretion and the expression of endothelial leukocyte adhesion molecules. Moreover, estrogen can induce apoptosis of cervical smooth muscle and promotes the binding of relaxin, which subsequently leads to an increase in HA synthesis and upregulation of MMP activity. However, with the cervix in mind, we believe that the main hormonal factor that regulates its maturation is progesterone. Its role is mainly to inhibit cervical ripening and maintain the pregnancy. However, during the final days of pregnancy, a physiological functional progesterone withdrawal occurs. When progesterone withdrawal occurs, there is a disinhibition of expression of factors such as IL-8, COX, MMPs, and NOS, and, subsequently, cervical remodeling can occur. This statement finds confirmation in studies that show that local application of progesterone antagonists leads to cervical ripening. There is ample evidence that progesterone withdrawal is linked with NF-κB action. Due to progesterone withdrawal, the NF-κB gains the ability to translocate to the nucleus, where it can bind to specific genes and induce the expression of proinflammatory cytokines. Taking the above into consideration, we suggest that progesterone withdrawal is a key activator of the expression of factors that significantly influence cervical remodeling. The role of NF-κB, which seems to be a key mediator of inflammatory response in the cervical ripening process, requires special attention. NF-κB has the ability to upregulate the expression of factors crucial for cervical ripening, such as COX-2, IL-1, IL-8, and IL-6. In this paper, we show that NF-κB contributes to progesterone withdrawal and its action appears to be related to GR receptor levels. Additionally, it was shown that PGs can modulate the NF-κB activity. Taking the above into consideration, we believe that NF-κB appears to be a trigger for inflammatory factors activation and plays a pivotal role in regulating the inflammatory response. However, a thorough understanding of this phenomenon and determining the influence of other factors on NF-κB activity requires further research. Despite our great understanding of the cellular and biochemical events that occur during cervical ripening, the search for the factors that initiate and modulate this process continues. A comprehensive understanding of the entire process of cervical ripening seems to be crucial in the context of induction of labor. A greater knowledge could provide us with the means to help women who suffer from dysfunctional labor.

## Data Availability

Not applicable.
